# Demand and unmet need for modern contraception among mothers from a Pelotas Birth Cohort

**DOI:** 10.11606/s1518-8787.2023057004863

**Published:** 2023-07-18

**Authors:** Gbènankpon Mathias Houvèssou, Simone Farías-Antúnez, Andréa D. Bertoldi, Mariângela Freitas da Silveira

**Affiliations:** I Universidade Federal de Pelotas Postgraduate Program in Epidemiology Pelotas RS Brazil Universidade Federal de Pelotas. Postgraduate Program in Epidemiology. Pelotas, RS, Brazil.

**Keywords:** Contraceptive Agents, Needs and Demand, Family Planning Services, Contraception

## Abstract

**OBJECTIVE:**

To determine the total demand and unmet need for contraception with modern methods and their determinants among mothers participating in the 2015 Pelotas Birth Cohort.

**METHODS:**

Data from the 48-month follow-up of mothers participating in the 2015 Pelotas Birth Cohort were analyzed. Only biological mothers (aged up to 49 years) of children belonging to the 2015 Birth Cohort and who answered the 48-month questionnaire were included in the study sample. Logistic regression and respective 95% confidence intervals were used to determine associated factors.

**RESULTS:**

The study sample consisted of 3577 biological mothers. The prevalence of use of any contraceptive and of modern contraceptives was 86.0% (95%CI: 84.8–87.1) and 84.9% (95%CI: 83.7–86.1), respectively. The prevalence of unmet need for modern contraceptives was 10.7% (95%CI: 9.7–11.7), and the total demand for contraceptives was 95.6%. The factors associated with an unmet need for modern contraception were being over 34 years of age (OR = 0.6, 95%CI: 0.5–0.8), not having a husband or partner (OR = 1.9, 95%CI: 1.4–2.6), not being the head of the household (OR = 0.6, 95%CI: 0.4–0.9), having had three or more pregnancies (OR = 1.9, 95%CI: 1.3–2.6), and having had an abortion at least once after the birth of the child participating in the cohort (OR = 1.9, 95%CI: 1.0–3.6).

**CONCLUSIONS:**

Despite the high prevalence of modern contraceptive use, one in ten women had an unmet need for modern contraception and was at risk of unplanned pregnancy.

## INTRODUCTION

Family planning and reproductive health programs significantly improve maternal and child health by reducing unplanned pregnancies and by potentially reducing maternal and child mortality^1^. One indicator to measure family planning is the unmet need for contraception. According to the World Health Organization (WHO), women with an unmet need for contraception are those who are fertile and sexually active but do not use any modern contraceptive method and report not wanting more children or wanting to postpone the next birth^2^.

An unmet need for family planning (FP) reveals problems with supply and demand for FP resources that might have serious implications for women, children, families, and society as a whole^3^. Reducing the unmet need for FP would significantly reduce unplanned pregnancy, unsafe abortion, short inter-pregnancy intervals, and pregnancies at an early age^4,5^.

Globally, in 2019, among women of childbearing age with a need for family planning, 270 million had an unmet need for modern contraceptive methods^6^. In low- and middle-income countries, the unmet need among married women ranges from 8% in Colombia to 38% in São Tomé and Príncipe, with countries such as Haiti, Ghana, and Uganda also recording high prevalence^7^. In Brazil, data from the 2013 national health survey, showed that the prevalence of modern contraceptives use by women in childbearing age was 79.4%^8^. Also, in a study carried out in 2019 with data from the 2006 *Pesquisa Nacional de Demografia e Saúde da Criança e da Mulher* (PNDS – National Survey on Demography and Health of Women and Children), the prevalence of unmet need for modern contraceptives was 8.3%^9^ (4.1% to space out pregnancies and 4.2% to limit them), and the poorest women were 6 times more likely to require methods to limit pregnancies when compared with the richest^9^.

Whereas many studies have been conducted on the unmet need for modern contraceptive methods in low- and middle-income countries, in Brazil, only two studies^9,10^ assessing the unmet need were found, using data from the PNDS from 1996^11^ and 2006^12^. Furthermore, few studies^13,14^ have assessed the unmet need for contraception in both married and unmarried women of childbearing age, since single but sexually active women may also be at risk for unplanned pregnancies.

Modern contraceptives are defined as all hormonal or barrier contraceptives, the copper intrauterine device (IUD), and female and male sterilization^15^. In more detail, modern contraceptive methods are classified into intrauterine devices and systems, subdermal implants, contraceptive patches, oral contraceptives, condoms (male and female), injectable contraceptives, emergency contraceptive pills, diaphragms and cervical caps, spermicidal agents, vaginal rings, sponge, and sterilization^16^.

With the increase in utilization of modern contraceptives^17^, studies on the unmet need for modern contraception among women of childbearing age are needed to provide an updated panorama of this population. This study aimed to determine the total demand and unmet need for contraception with modern methods and their determinants in mothers participating in the 2015 Pelotas Birth Cohort.

## METHODS

For this study, data from mothers participating in the 2015 Pelotas Birth Cohort were analyzed. Pelotas is in the southern region of the state of Rio Grande do Sul, Brazil. According to an estimate from the Brazilian Institute for Geography and Statistics (IBGE), its population in 2020 was 343,132 inhabitants being the fourth largest city in the state^18^.

The 2015 Birth Cohort is a longitudinal study evaluating the determinants of newborn health and its changes over a lifetime. Mothers living in the urban region of Pelotas who were due to deliver in 2015 were interviewed during pregnancy. After the birth of their children, follow-ups were carried out at three, twelve, twenty-four, and forty-eight months. In the perinatal period, 4275 of the 4333 eligible children were interviewed. A detailed description of the 2015 Birth Cohort methodology is available elsewhere^19^.

Data from the 48-month follow-up were used for this study. They were electronically stored in the REDCap system (Research Electronic Data Capture)^20^. Mothers answered questions on different aspects of their children’s health, their own characteristics and health, and contraception, among others. Fieldwork for this follow-up began on January 7, 2019, and ended on December 31, 2019. All interviewers involved in the follow-up received training periodically.

Only biological mothers (aged up to 49 years) of children belonging to the 2015 Birth Cohort and who answered the 48-month questionnaire were included in the study sample. Interviews completed by the child’s biological father, grandmother, or any other person responsible for the child were excluded from the analyses. Also, responding mothers who were not sexually active, who did not respond to part of the questionnaire pertaining to pregnancy and contraceptive use, and those over 49 years of age were excluded from the analyses. Finally, for questionnaires answered twice by mothers of twins, only one of them was considered.

This study considered women with an unmet need for contraception to be those of childbearing age (age up to 49 years), married or not, who were sexually active and did not wish to have more children and/or had doubts as to whether and when they intended to become pregnant but were not using a contraceptive method^21,22^. Women pregnant at the time of the interview whose current/recent pregnancy was unplanned or mistimed were also classified as having an unmet need^22^, as were women who use other antiquated methods, such as an ovulation calendar.

The independent variables studied were: women’s age in years, family income in minimum wages, women’s education in years, having a husband or partner (Yes/No), watching TV frequently (Yes/No), being the head of the household (Child’s father/Child’s mother/Other); number of pregnancies (1–2/3 or more); number of stillbirths after the birth of the child participating in the cohort (None/At least one); and number of abortions after the birth of the child participating in the cohort (None/At least one). To assess the contribution of health services utilization to the unmet need for modern contraceptive methods, the following variables were included: having received medical advice on how to avoid pregnancy (Yes/No); possessing health insurance or a health plan (Yes/No); and the means of payment for hospitalization when the child was born (The Brazilian Unified Health System [SUS]/Health Insurance/Private). These variables were extracted from the 3-month follow-up after childbirth since they are not available in the 48-month questionnaire.

**Figure 1:**
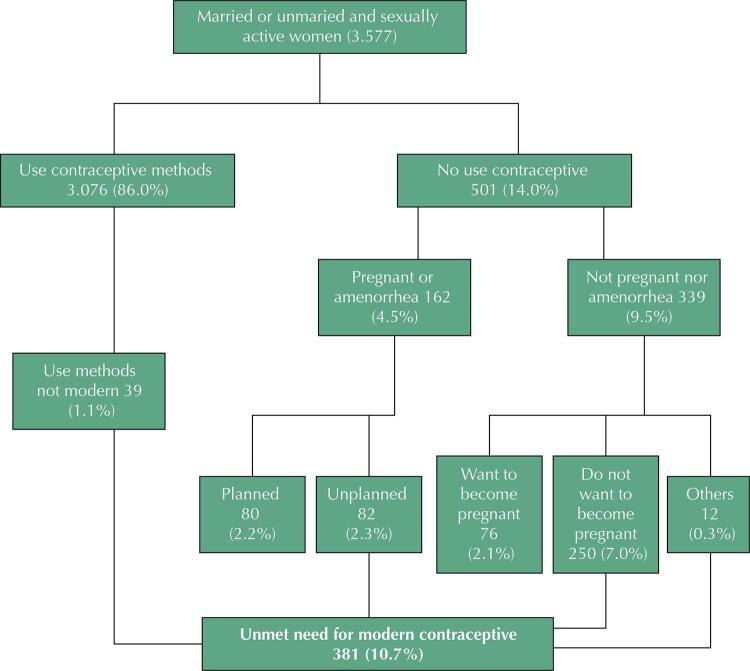
Percentage distribution of unmet need for modern contraception among women participating in the 2015 Pelotas Birth Cohort. 2022.

Source: Bradley et al.^22^

Statistical analysis was performed with Stata 14.0 (Stata Corp., College Station, Texas, USA). Descriptive analyses of the independent variables and the outcome were initially performed. Next, total demand for contraception was calculated. Total demand was the percentage of women with an unmet need plus the percentage of those with the satisfied need (proportion using modern contraceptives). Regarding the unmet need, it was determined following the calculation from Figure 1. Bivariate and multivariate analyses evaluated the association between the outcome and the independent variables by calculation of crude and adjusted odds ratios (OR) with logistic regression and of their respective confidence intervals (95%CI).

**Figure 2:**
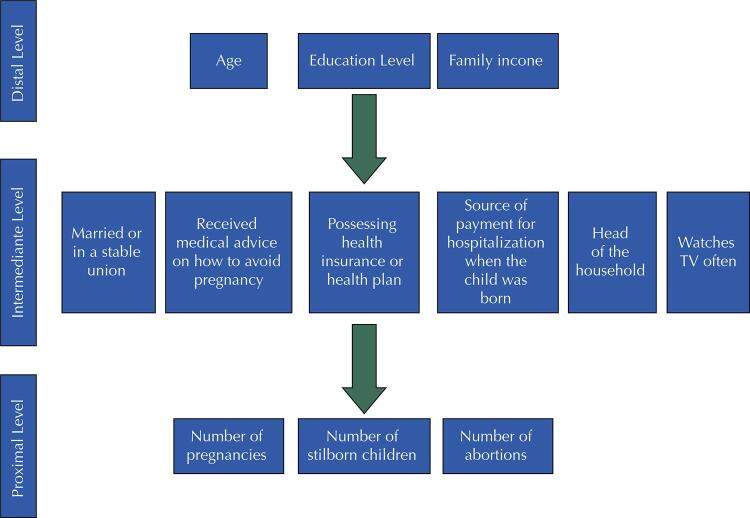
Conceptual model.

Variables for the multivariate models were selected by backward selection following a hierarchical conceptual model (Figure 2). The variables age, women’s education, and family income were at the most distal level. At the intermediate level were the variables having a husband or partner, receiving medical advice on how to avoid pregnancy, possessing health insurance or a health plan, means of payment for hospitalization when the child was born, watching TV frequently, and being head of the household. Finally, at the proximal level were the following variables: number of pregnancies, number of stillbirths after birth of the child participating in the cohort, and number of abortions after birth of the child participating in the cohort. Variables with a p-value lower than 0.2 remained in the model to control for confounders. This value was chosen to consider variables that may be a potential confounders^23^. And a significance test p-value lower than 0.05 was considered. Wald’s test was used as a guide for selecting variables from the multivariate models, and a likelihood ratio test was used to estimate the p-value of each variable.

## RESULTS

**Figure 3:**
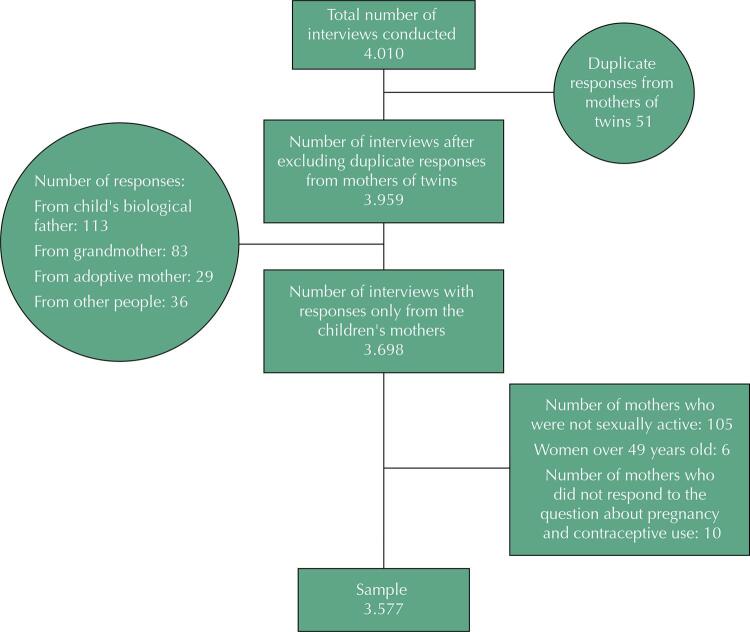
Sample selection flowchart.

In the 48-month follow-up, 4,010 interviews were carried out, with 3,959 without duplicates considering mothers of twins. Of these, 261 interviews answered by the child’s biological father and people other than the biological mother were excluded. After excluding from the questionnaire mothers who were not having sexual intercourse at the time of the interview, those aged over 49 years, and those without an answer about pregnancy and contraception (125 interviews), the studied sample consisted of 3,577 biological mothers (Figure 3).

Mothers excluded for not meeting the inclusion criteria represent 6.4% of the mothers of children participating in the birth cohort. These non-interviewed women were younger (10.5% under the age of 25) and less educated (17.4% with education between 0 and 5 years) than those included in the study.

The average age of the women studied was 31 years (standard deviation: 6.5 years), with 56.2% being 30 years of age or older. Nearly half (47.6%) had a family income between 1 and 3 minimum wages and 66.5% had less than 12 years of schooling (Table 1). Most were married (82.6%) and 91.9% had between 1 and 2 children. The prevalence of use of any contraceptive and of modern contraceptives was 86.0% (95%CI: 84.8–87.1) and 84.9% (95%CI: 83.7–86.1), respectively. The prevalence of unmet need for contraceptives was 10.7% (95%CI: 9.7–11.7) (Figure 1), and the total demand was 95.6%.

**Table 1:** Characteristics of biological mothers participating in the 2015 Pelotas Birth Cohort. (n = 3,577).

Variables	n	%
Age (years)		
< 25	693	19.4
25–29	873	24.4
30–34	882	24.7
≥ 35	1,129	31.5
Family income in minimum monthly wages (BRL 998.00)		
< 1	382	10.8
1.1–3.0	1,689	47.6
3.1–6.0	944	26.6
6.1–10	271	7.7
> 10	258	7.3
Education level		
0–5	285	8.0
5–9	1,023	28.6
10–11	1,069	29.9
≥ 12	1,2	33.5
Married or in union		
No	623	17.4
Yes	2,952	82.6
Receipt of medical advice on how to avoid pregnancy		
No	1,503	42.7
Yes	2,017	57.3
Possession of health insurance or health plan		
No	1,909	54.2
Yes	1,614	45.8
Source of payment for hospitalization when the child was born		
SUS	2,412	68.4
Healthcare insurance	753	21.4
Private	359	10.2
Watches TV often		
No	743	20.8
Yes	2,825	79.2
Head of the household		
Child´s father	1,765	51.2
Child´s mother	1,250	36.3
Other	431	12.5
Number of pregnancies		
1–2	3,288	91.9
≥ 3	289	8.1
Number of stillborn children after birth of the child participating in the Cohort		
None	3,534	98.8
At least one	43	1.2
Number of induced abortions or miscarriages after birth of the child participating in the Cohort		
None	3,509	98.1
At least one	68	1.9

Table 2 shows the crude and adjusted analyses of factors related to an unmet need for contraceptives. In the multivariate analysis, as age increases, the chance of a mother having an unmet need for contraception decreases, with mothers older than 34 years showing 40% (OR = 0.6, 95%CI: 0.5–0.8) less odds of having an unmet need compared with younger mothers. Women without a husband or partner were 1.9 (95%CI: 1.4–2.6) times more likely to have an unmet need for contraception than those who were married or had a partner. Those who identified the head of the household as the child’s father or someone else had lower chances of having an unmet need for contraception than mothers who were heads of the household (OR = 0.9, 95%CI: 0.7–1.1 and OR = 0.6, 95%CI: 0.4–0.9, respectively). Regarding the number of pregnancies, mothers who became pregnant three or more times were 90% (OR = 1.9, 95%CI: 1.3–2.6) more likely to have an unmet need for contraception than those who became pregnant once or twice. Also, having an abortion at least once after the birth of the child participating in the cohort increased the chances of needing contraception 1.9 times (95%CI: 1.0–3.6). Family income (p = 0.130), education level (p = 0.117), having received medical advice on how to avoid pregnancy (p = 0.583), possessing a health insurance or health plan (p = 0.389), the source of payment for hospitalization when the child was born (p = 0.766), and watching TV (p = 0.803) were not associated with an unmet need for contraception.

**Table 2:** Crude and adjusted analysis of factors associated with an unmet need for modern contraception in mothers participating in the 2015 Pelotas Birth Cohort.

Variable	Prevalence of unmet need		Crude analysis			Adjusted analysis		
		
	%	95%CI	OR	95%CI	p-valor*	OR	95%CI	p-valor*
Age (Years)					0.002			0.008**
< 25	14.6	12.1 – 17.4	Ref..			Ref..		
25–29	10.9	9.0 – 13.1	0.7	0.5–0.9		0.8	0.5–1.0	
30–34	9.0	7.2–11.0	0.6	0.4–0.8		0.6	0.4–0.8	
≥ 35	9.4	7.8–11.3	0.6	0.5–0.8		0.6	0.5–0.9	
Family income in minimum monthly wages (BRL 998.00)					0.007			0.130
< 1	14.1	11.0–18.1	1.9	1.3–2.8		1.5	1.1–2.4	
1.1–3.0	11.6	10.2–13.2	1.5	1.2–2.0		1.4	1.0–1.9	
3.1–6.0	7.9	6.4–9.9	Ref..		Ref..			
6.1–10	10.7	7.5–15.0	1.4	0.9–2.2		1.4	0.9–2.3	
> 10	9.7	6.6–14.0	1.2	0.8–2.0		1.3	0.8–2.1	
Education level in years					0.007			0.117
0–5	16.5	12.6–21.3	Ref..			Ref..		
5–9	11.3	9.6–13.5	0.6	0.4–0.9		0.6	0.4–0.9	
10–11	9.7	8.1–11.7	0.5	0.4–0.8		0.6	0.4–0.9	
≥ 12	9.5	8.0–11.3	0.5	0.4–0.8		0.7	0.5–1.0	
Married or in a stable union					< 0.001			< 0.001
No	16.4	13.7–19.5	1.9	1.5–2.4		1.9	1.4–2.6	
Yes	9.4	8.4–10.5	Ref.			Ref.		
Received medical advice on how to avoid pregnancy					0.583		-	
No	10.4	8.9–12.0	Ref.					
Yes	11.0	9.7–12.4	1.1	0.9–1.3			-	
Possessing health insurance or health plan					0.398		-	
No	11.1	9.8–12.6	Ref.					
Yes	10.2	8.8–11.8	0.9	0.7–1.1			-	
Source of payment for hospitalization when the child was born					0.158			0.766
SUS	11.2	10.0–12.6	1.4	0.9–2.1		1.1	0.7–1.8	
Healthcare insurance	10.2	8.3–12.6	1.3	0.8–2.0		1.2	0.7–1.9	
Private	8.1	5.7–11.4		Ref.		Ref.		
Watches TV often					0.803		-	
No	10.9	8.9–13.4	Ref.				-	
Yes	10.6	9.5–11.8	0.9	0.7–1.3			-	
Head of the household					0.007			0.047
Child´s father	9.1	7.9–10.6	0.7	0.5–0.9		0.9	0.7–1.1	
Child´s mother	12.7	11.0–14.7	Ref.			Ref.		
Other	10.4	7.9–13.7	0.8	0.6–1.1		0.6	04–0.9	
Number of pregnancies					< 0.001			0.001
1–2	9.9	8.9–11.0	Ref.			Ref.		
≥ 3	19.0	14.9–24.0	2.1	1.6–2.9		1.9	1.3–2.6	
Number of stillborn children after the birth of the child participating in the cohort					0.261		-	
None		10.6	9.6–11.7	Ref.		-	-	
At least one		16.3	7.7–31.1	1.6		-	-	
Number of induced abortions or miscarriages after the birth of the child participating in the cohort								0.047
None	10.4	9.4–11.5	Ref.			Ref.		
At least one	23.5	14.8–35.3	2.7	1.5–4.7		1.9	1.0–3.6	

*Likelihood ratio test

**Linear trend test

OR: odds ratio; 95%CI: 95 % confidence interval.

## DISCUSSION

This study aimed to estimate the prevalence of the use of and the unmet need for modern contraception and their determinants, finding that three out of four mothers use modern contraception and one in ten has an unmet need for modern contraception. Being younger, not having a partner, being the head of the household, having a higher number of pregnancies and a higher number of abortions after the birth of the child participating in the cohort were all associated with an unmet need for contraception.

De Leon et al.^8^ reported the prevalence of use of any contraceptive and of modern contraceptives in women of childbearing age in Brazil as 82.0% and 79.4%, respectively, which is lower than what we found. This could be due to the study’s sample only including married women or women in a stable union. Furthermore, the difference could be due to the assessment of women that had already given birth (mothers), exclusively, in this study.

In a study using data from the 2006 National Survey on Demography and Health of Women and Children (PNDS), Carvalho^9^ found a prevalence of unmet need for contraception of 8.3% in Brazil. Using data from that same national survey in 1996, Tavares et al.^10^ found an unsatisfied demand for contraception of 7.3% among married/in-law women, suggesting an increase over the years. Furthermore, this study included single mothers who had the greatest unmet need for contraception, and studied only mothers, which may explain the higher prevalence. The estimates of unmet need of studies from Egypt, studying women one year after giving birth^24^, and from Ethiopia, evaluating women who had children in the previous five years before the interview date^25^, were 14.9%^23^ and 22.0%^24^ respectively, exceeding that found in our study. Bishwajit et al^4^. Found, in Bangladesh, a slightly higher percentage of unmet need (13.5%) than on our study. Studies carried out in low-income countries^25-28^ found a higher unmet need for any method compared with the United Nations cutoff point, which considers the prevalence of unmet need for any contraception to be high at 20%^29^.

Although the estimated prevalence of unmet need of this study is not high, these women are at risk for unwanted pregnancies. Sully et al.^30^ estimated that women with an unmet need for modern contraception are responsible for 77% of all unwanted pregnancies. Therefore, improving access to contraceptive methods and reducing the unmet need are the primary objectives of FP programs and policies and are essential to achieving global public health goals such as the Sustainable Development Goals on sexual and reproductive health^31^.

Among associated factors in the multivariate analysis, age stood out. The older a woman is, the less likely she will have an unmet contraceptive need. However, Deyessa et al.^25^, assessing women who had children in the five years before the interview date, reported women from 35 to 49 years old had greater chance of having an unmet need. Other studies, despite evaluating women of childbearing age in general, found that younger women were more likely to have an unmet need for modern contraception^32,33^. These findings can be explained by the inconsistent use of modern methods, a problem that young women experience more often^34^.

Women without a husband or partner but who are sexually active had a greater unmet need for modern contraception. A 2018 study by Juarez et al.^13^ carried out in Mexico, which evaluated childbearing age women in general, found that women who had never been in a stable union had a greater unmet need both for spacing and for limiting pregnancies. This finding could be explained by the women and their partners having the opportunity to discuss the issue of family planning. The literature points out that discussing family planning between a couple reduces the chance that the woman will have an unmet need for a modern contraceptive method^35,36^. The role of gender and other social factors related to FP communication and decision-making play essential roles in adopting and continuing the contraceptive method^37^. In our study, women who considered the child’s father or someone else as head of the household were less likely to need a modern method than those who saw themselves as such. The woman head of the household would consider herself independent and would have less support or less access to contraception due to lower economic power.

Another factor associated with unmet need was the number of pregnancies: women with three or more pregnancies had a greater unmet need for contraception using modern methods. Similarly, Solomon et al.^38^ reported that multiparous women had a greater need for contraception, despite assessing women of childbearing age in general. A study with a sample of Brazilian women found that the greater the number of children, the greater the demand not met by any method of contraception^9^, corroborating our findings. The increased number of children may be a result of previous unmet need, leading to one or more unplanned pregnancies.

Family income, education level, and watching TV were not associated with an unmet need for contraception. However, studies assessing postpartum women reported that being richer and watching TV were associated with reducing the unmet need for contraception in Indonesia^26^, whereas women with lower education level were more likely to have an unmet need in Ethiopia^27^.

Knowing whether the abortions recorded in the 2015 cohort were induced or whether they were miscarriages was impossible, since this information was not available. This is one of the limitations of the study, making it difficult to form conclusions from this result. Note other limitations in this study. The three variables related to use of health services included in the analyses (receiving medical advice on how to avoid pregnancy, possessing health insurance or a health plan, and the means of payment for hospitalization when the child was born) were used as a proxy; they were collected after a three-month follow-up. Therefore, they may not have represented the current situation of some respondents at the 48-month follow-up. Among the women who were not interviewed, the highest proportion was younger than 25 years old, and this study found that young women were more likely to have an unmet need for contraception than those aged 35 or over. Thus, the prevalence of unmet need for contraception could be underestimated. We found few studies evaluating married and single women in the literature, and most studies used to discuss the findings are those that just studied women who were married or in a stable union. Thus, comparing our study to those studies used for discussion, the prevalence of unmet need may be overestimated since single women were more likely to have an unmet need. Another methodological difference was that most of these studies were carried out with a representative population sample whereas the sample of our study was of women participating in a birth cohort from a city. This difference in sampling could overestimate the prevalence of contraceptive use and of unmet need reported in our study. Furthermore, an important limitation is that the results of this study cannot be generalized for all women in childbearing age, since it only evaluated mothers.

Reducing or eliminating the unmet need for contraception offers opportunities to reduce the burden of unintended pregnancies and associated abortions in developing countries, where they are more likely to be unsafe and associated with death^39^.

## CONCLUSION

Despite the high prevalence of modern contraceptive use among mothers, some mothers are still at risk of unwanted pregnancy due to an unmet need for modern contraception. This can lead to complications related to the health of the woman and the child. Policies and programs in family planning and women’s health must be strengthened to ensure reproductive health rights.
